# Serum Therapy in Tuberculosis

**Published:** 1908-11

**Authors:** Louis C. Rouglin

**Affiliations:** Ex-Vice President American Anti-Tuberculosis League, Secretary Georgia Anti-Tubercu-Losis League, Member American Medical Association, Secretary Southern Sanitarium Association, Physician to Pine Ridge Sanitarium for Tuberculosis, etc., Atlanta, Ga.


### SERUM THERAPY IN TUBERCULOSIS.

BY DR. LOUIS C. ROUGLIN, EX-VICE PRESIDENT AMERICAN ANTI-TUBERCULOSIS LEAGUE, SECRETARY GEORGIA ANTI-TUBERCULOSIS LEAGUE, MEMBER AMERICAN MEDICAL ASSOCIATION, SECRETARY SOUTHERN SANITARIUM ASSOCIATION, PHYSICIAN TO PINE RIDGE SANITARIUM FOR TUBERCULOSIS, ETC., ATLANTA, GA.

*Synopsis of Subjects.*—Tuberculin vs. Drug Therapy—Method of Preparations—Mode of Action—Report of Cases—Method of Administration—Conclusion.

Science has demonstrated two facts, which are of supreme importance to the millions of people who live in daily dread of "The Great White Plague." These facts are:

First—That tuberculosis is a preventable disease.

Second—That of all grave diseases, pulmonary tuberculosis, in its incipiency, is the most easily cured.

Drug therapy, however, having no influence on the bacilus, has proven an absolute failure, and in most cases has done more harm than good. The powers of digestion, assimilation and nutrition is the stronghold of the consumptve. Derange these factors and the patient is lost. What, therefore, can be more injurious than the indiscriminate drugging of the consumptive with mercury, arsenic, creosote, guiacol, cough-syrups and the like, when instead of improving the appetite, in the bulk of cases it destroys what little appetite the patient had. RUIN HIS DIGESTION, AND THE PATIENT IS DOOMED.

Hygienic and dietetic care does all the good that can be desired for the general condition, and for the specific tubercule, Serum Therapy, offers the only hopeful, reliable and efficient solution for the condition.

*Methods of Preparation.*—As a prelude, I believe it to be of interest to review briefly the various steps made in the methods of the different preparations of tuberculin, given in detail, such preparations only, that are most commonly used, and briefly showing the progress made in its preparation from the time of its discovery by Koch in 1890 to the present date.

Tuberculin Old as prepared originally by Koch is as follows: Transfer Tubercule bacilli from a pure culture into a flask containing sterile glycerine bouillon (beef extract 1 per cent., pepton 1 per cent., glycerine 5 per cent.) and allow to grow from 6 to 8 weeks. Concentrate the contents of the flask by boiling to one-tenth its original bulk and filter. The filtrate represents ten per cent. extract, ten per cent, peptons and fifty per cent. glycerine in water.

In 1891, Hunter, of London, removed the beef extract, peptons and glycerine by precipitating the tuberculin with platinum chloride, which carries down albumoses and peptons and used the filtrate.

In 1893, Klebs precipitated Koch's tuberculin with sodic iodide of bismuth, then further precipitating the filtrate with absolute alcohol, collecting the residue and re-dissolving in water, obtaining one per cent. organic substance in solution.

In 1894, Klebs used only the filtered glycerine bouillon, upon which the tubercule bacilli had been grown, avoiding boiling heat.

In 1896, Von Ruck boiled in a vacuum the whole culture (tubercule bacilli and glycerine bouillon) from 4 to 6 weeks at 130 degrees F., with the exception of extracting more toxins from the germ. Objections raised to these various methods are: Heat used that is likely to injure the toxins, the uncertainty of the amount of toxins, the presence of the glycerine, beef extract and bouillon, which is very irritating hyperdermically.

Koch's New Tuberculin T. R. was announced by Koch in April, 1897, and is prepared from bacilli of a virulent culture, dried in a vacuum in the dark and pulverized. The powder is then put in distilled water and centrifuged at a high speed for about forty-five minutes. The upper clear liquid consisting of fat and debris is decanted and discarded. The precipitate is again dried in a vacuum in the dark, again suspended in water, and centrifuged, and the residue is again treated as before. This process is repeated several times, and the residue finally obtained is known as Tuberculin Ruckstande or T. R.

In May, 1897, Von Ruck announced a preparation from the bodies of Tubercule bacilli, which are first washed, then dried and powdered. The fat is then extracted with alcohol and ether. The residue is then dried and powdered again and finally extracted in distilled water.

More recently Koch prepared an emulsion which consists of a suspension of ground up Tubercule bacillin 50 per cent. glycerine so that each c. c. contains 5 mg. of the powder.

*Objections.*—In all the latter preparations the objection to heat is eliminated. The possibility of infecting the patient with a virulent bacillus is considered as a disadvantage. This, however, is guarded against by careful bacteriological examination, or by heating to 60 degrees C. for two hours.

*Mode of Action.*—Tuberculin when injected beneath the skin excites an inflammatory reaction, which manifests itself by fever, malaise, nausea and even vomiting, while locally, we get Rales. If the dose be too large, or, regardless of reaction if the injections are continued, necrosis of the tubercular areas, cavity formation and increased infiltration at the periphery occurs, but when administered in very small doses and increased cautiously so as to avoid febrile reactions, it is asserted that the local inflammatory changes lead to formation of fibrous tissue, which shuts off the Tuberculosis foci. Dr. Julien Citron explains the action of Tuberculin as follows: The Tubercule bacilli or a portion of their body substance, in an unknown manner, unite with the receptors of the bordering cells; the cell receptors so attacked are over-compensated by the cell, and as a result we may distinguished four stages.

First Stage—An increase of the reciptors (antibodies) attached to the cell.

Second Stage—Increase of the attached receptors and the presence of free antibodies in the foci of infection.

Third Stage—A great number of attached receptors with a dimunition of the free antibodies. In all three stages there exists a specific hypersensibility to tuberculin. When tuberculin is introduced in small amounts into the organism, there is no reaction by the normal cells, but the cells provided with receptors for tuberculin in and around the foci of infection, are attacked and combine with tuberculin, producing the reaction which is characterized by fever and other symptoms. The cells so attacked immediately produce great numbers or receptors (antibodies) to compensate for those attached by tuberculin. There occurs under the influence of tubercule bacillus preparations (tuberculins) a new formation of aglutinins, anti-tuberculin and opsonins.

Fourth Stage—After long continued injections of small amounts of tuberculin, anti-tuberculin is found in the serum, and this brings us to the fourth stage in which there are many fixed receptors, and also a large number of free antibodies at the point of local infection and in the serum. To reach this stage tuberculin must be injected over a considerable length of time, and in sufficient quantities to stimulate the cells to the production of anti-tuberculin and not to the point of intolerance or toxic-effects.

*Report of Cases.*—From a series of 39 cases of Tuberculosis which have been under my observation and treatment during the past three years, the following selected cases will serve as an illustration as to the progress made under the administration of tuberculin. Of this series 19 are completely cured six months or more, eleven have made marked improvement and are not now under treatment. Eight are improved and are now continuing treatment, one died during treatment with acute pneumonia. In this series are not included cases which were seen too late, and when the use of serum was not advisable.

Case 1—G. W., male, age 28, was advised by a New York physician to go South on account of his lungs. Gave a history of night sweats, rapid loss of weight, and several small hemorrhages from the lungs. Tubercule bacilli found in the sputum by New York Board of Health. Patient coughed incessantly, runs an evening temperature of 101 degrees to 101.5 degrees F., weighs 127 pounds, physical sign were those of marked consolidation of upper right lobe, and to a lesser degree at the upper and lower lobe of left side. Injections of tuberculin began on 14th day after first examination, and continued for 22 weeks, cough and fever disappeared entirely, and repeated examinations of the sputum fail to reveal any T. B. and all abnormal signs of chest have completely cleared up. Patient now weighs 157 pounds, and is actively engaged in the mercantile buiness in Atlanta.

Case 2—Mrs. W. T. C., referred to me by Dr. George Brown, age 32, gave history of three successive attacks of pneumonia, had grippe four months ago, continued coughing and lost 18 pounds. Had several small hemorrhages from the lungs; evening temperature ran as high as 103 degrees F. Tubercule bacilli present in the sputum. Physical examination revealed diseased condition of practically over the whole of both lobes of left side, and pleurisy on both sides. Tuberculin began six weeks after first seen, cough and night sweats disappeared after fifth week of injections, appetite improved and patient gained in weight. Treatment continued for 8 months. Patient feels absolutely well. T. B. are yet found in the sputum in small numbers, and some of the physical signs are still present. When last seen, more than eight months after last treatment, patient claimed to feel perfectly well, and on that account refused further treatment.

Case 3—I. S. male, age 42, had pleurisy on right side a year ago, was confined to bed three weeks, was never relieved entirely of the pain, had to go to bed again six months ago for two weeks, and has been coughing and felt sick ever since. Cough worse at night and early morning. Had four severe hemorrhages from the lungs, has night sweats and feels very weak and lost some flesh. Sputum did not show any T. B. On consultation with Dr. Todd, patient went to Asheville, but returned in six weeks with all the symptoms aggravated. Physical signs showed evidence of marked consolidation of upper right lobe and numerous Tubercule bacilli were now found in his sputum. Treatment continued for five and a half month, and now. over two years since his last injecton patient holds his usual weight, is attending to his duties as manager for a department store, and shows absolutely no evidence of the disease.

Case 4—J. R. male, age 19, referred to me by Dr. George Brown, gave history of eight months rapidly failing in health, night sweats, expectoration and troublesome cough. Lost 18 pounds in weight, hoarse, pulse 120, temperature 102. Appetite poor, complains of heart burn, indigestion, and occasional vomiting, of food. Physical examination shows involvement of both apices, that of the right being more extensive. Patient was under treatment for 12 months, and while at first he seemed to decline, later, however, he made steady progress and finally improvement became rapid. He is now conductor on the street cars of Atlanta, and when last seen looked robust(, and has grown rather stout, and says he has not had the slightest sign of illness in the last 12 months.

*Method of Administration.*—Under no circummstances do I institute any form of treatment until I have the patient under complete observation for at least 24 hours, having his temperature, pulse and respiration taken and recorded every three hours. Also the condition of his bowels, kidneys, etc., properly observed. I then prescribe such measures as the condition may indicate, laxatives or purgatives as the case may demand. No drugs for the cough unless same is very troublesome. Cold sponging for the night sweats, or a sponge with alum water. Drugs only when these fail and the sweats are excessive and severe. For the temperature, my rule is, that whenever my patient shows a rise of temperature, of, even as low as one-half a degree, he must rest, and rest in bed. As to diet, I give my patients food at frequent intervals. Food such as is found by observation in each individual case, that the patient can best digest and assimulate, raw eggs, milk, cream. Keffir in such quantities as can best be borne by the patient.

The injections of tuberculin is not given until the patient has been free from abnormal temperature for 24 hours, this rule is absolute, and one to which I have so far made no exceptions. I begin the initial dose with 1-1000 gm. and increase the dose gradually and cautiously until my patient gets 1 1-4 gm. without producing any reaction. Under no circumstances do I give another injection until all local and constitutional signs of the reaction, if any, have disappeared. Under no circumstances do I increase a dose, if a lesser dose shows any signs of producing a reaction. Under no circumstances do I give an injection if the patients' condition is below his usual standard from any cause.

Slight digestive disturbances, headaches, malaise calls for an interval of rest from injections, and the next injection is not given until all those minor symptoms have been overcome. By following this method we may produce tuberculin immunity in a patient without causing marked reaction or any disturbance of the patient's general health.

The use of the opsonic index as a control for serum therapy in tuberculosis has many advocates, while a good number of competent observers do not consider it necessary or advisable. So far I cannot speak from personal experience on the subject, but at the Pine Ridge Sanitarium, where I have the advantage of making careful and accurate observations, I am now having taken and recorded, the opsonic index of every patient treated, and as soon as a sufficient number of observations have been made, over a suitable length of time, I shall give a report to the Society of the results of the opsonic index in relation to tuberculosis as observed at the Pine Ridge Sanitarium.

*Conclusion.*—In conclusion I wish to state my belief that in tuberculin we possess a remedy for tuberculosis, which may be justly considered a specific, at least in the earlier stages of the disease, and the adverse reports, made by earlier observers, were due to faulty methods, of administration, and an improper conception of its use. When properly employed, one is forced to admit that in tuberculin, we at least possess a remedy which is far superior and accomplishes more good than any other drug known. But in order to obtain the best results from its use, it must be given by competent men, and should be combined with proper hygienic and dietetic treatment. We also must not forget that it is a remedy which possesses great dangers, as well as great virtues—that the utmost amount of care and caution is necessary to obtain the best results, and that in the hands of the careless and inexperienced it may bring ruin and disaster.

829-30 Candler Building, Atlanta, Ga.

